# Sustainability of Heating, Ventilation and Air-Conditioning (HVAC) Systems in Buildings—An Overview

**DOI:** 10.3390/ijerph19021016

**Published:** 2022-01-17

**Authors:** Nilofar Asim, Marzieh Badiei, Masita Mohammad, Halim Razali, Armin Rajabi, Lim Chin Haw, Mariyam Jameelah Ghazali

**Affiliations:** 1 Solar Energy Research Institute, Universiti Kebangsaan Malaysia, Bangi 43600, Malaysia; masita@ukm.edu.my (M.M.); drhalimrazali@ukm.edu.my (H.R.); chinhaw.lim@ukm.edu.my (L.C.H.); 2 Independent Researcher, Razavi 16, Mashhad 91777-35843, Iran; gbadiei317@gmail.com; 3 Department of Mechanical and Manufacturing Engineering, Faculty of Engineering and Built Environment, Universiti Kebangsaan Malaysia, Bangi 43600, Malaysia; arminukm50@siswa.ukm.edu.my

**Keywords:** HVAC systems, sustainability, energy efficient, indoor air quality, water recovery, retrofitting

## Abstract

Increasing demand on heating, ventilation, and air-conditioning (HVAC) systems and their importance, as the respiratory system of buildings, in developing and spreading various microbial contaminations and diseases with their huge global energy consumption share have forced researchers, industries, and policymakers to focus on improving the sustainability of HVAC systems. Understanding and considering various parameters related to the sustainability of new and existing HVAC systems as the respiratory system of buildings are vital to providing healthy, energy-efficient, and economical options for various building types. However, the greatest opportunities for improving the sustainability of HVAC systems exist at the design stage of new facilities and the retrofitting of existing equipment. Considering the high available percentage of existing HVAC systems globally reveals the importance of their retrofitting. The attempt has been made to gather all important parameters that affect decision-making to select the optimum HVAC system development considerations among the various opportunities that are available for sustainability improvement.

## 1. Introduction

Population growth, modern technologies, and lifestyles are among the reasons for the necessity of heating, ventilation, and air conditioning (HVAC) systems in various types of buildings. Meanwhile, HVAC systems have an important role in the comfort and safety of indoor air quality (IAQ). However, these systems account for 40–60% of energy usage in buildings [1] or 15% of the world’s total energy consumption (Rafique 2018). These facts illustrate the importance of considering HVAC sustainability development by researchers, industries, and policymakers. Furthermore, sustainability considerations and innovations in HVAC systems are necessary to provide a remarkable, healthy, productive, and sustainable built environment for occupants while reducing energy consumption and costs [2].

For the sustainable development of all three pillars, namely, economic, environmental, and social, reaching the immediate and long-term benefits of people, the planet, and prosperity must be carefully considered [3].

**Figure 1:**
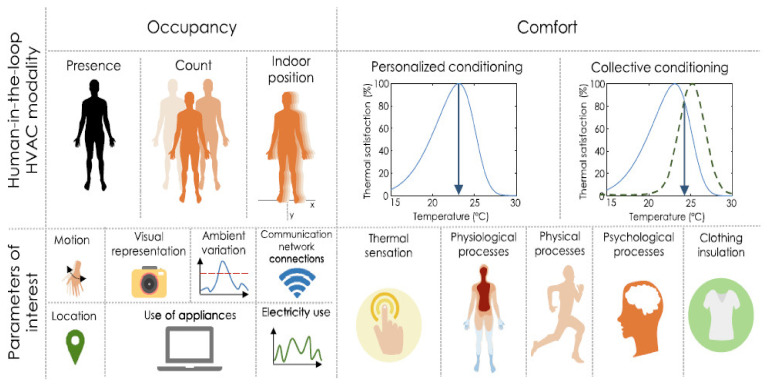
Human-in-the-loop HVAC modalities of occupancy and comfort along with their parameters of interest [4].

Various affecting parameters must be considered to improve the sustainability of HVAC systems. A remarkable understanding of human-in-the-loop HVAC systems could facilitate and improve the achievement of sustainability (Figure 1) [4]. Considering all the related parameters will help minimize energy usage without compromising occupant thermal comfort. 

The assessment of measurement and modeling techniques and their performance are important in planning to achieve sustainable HVAC systems considering occupancy, comfort, and building type [4].

Despite the availability of various methods that can be used in building design and retrofitting to improve the HVAC system performance [5,6], this paper only studies the parameters that directly deal with HVAC sustainability improvement. However, many of these parameters, such as energy, environment, and water recovery, overlap with one another.

## 2. Energy

The high energy share of HVAC systems in buildings, which increase greenhouse gas emissions and costs, motivates researchers to study the various parameters related to reducing energy consumption and finding sustainable solutions for HVAC systems [7,8]. Many studies have been conducted to develop advanced design and control strategies to optimize energy, thermal, and environmental performance and to make these strategies cost-effective for HVAC systems [2,9,10,11].

Renewable energies could improve the sustainability of HVAC systems directly or indirectly. Considering the climate and geographical conditions, the use of various heating and cooling technologies that utilize renewable energy sources in HVAC systems in the building are very important options in improving sustainability.

Desiccant heating, cooling, and ventilation [12,13,14,15]; evaporative passive cooling [16,17]; solar heating and cooling systems [18,19]; geothermal heating and cooling systems [20]; and biomass heating and cooling technologies [21,22] are examples of renewable technologies that are used in HVAC systems in buildings.

Various HVAC system designs that utilize renewable energy sources have been developed and explored [23,24]. Ma et al. [2] studied the HVAC systems in solar-powered decathlon houses developed in the USA. They found that most of them used heat pumps for space heating and cooling. After 2005, more than half of houses utilized energy/heat recovery ventilators for improving the HVAC performance. Meanwhile, various other technologies such as phase change materials, night-time radiative cooling, evaporative cooling, and desiccant dehumidification are employed for electrical energy saving in HVAC systems.

Modified and improved designs for various components in HVAC systems, such as dampers dampers [25,26], filters [27,28], humidifier, dehumidifier [29,30], heating and cooling coils [31], and ducts and fans [27,32], can reduce the energy consumption of such systems while optimizing their performance [13].

Waste heat and energy recovery are important for reducing energy usage in HVAC systems [33,34,35,36].

Air-to-air heat exchangers [37] and heat pipe heat exchangers (HPHE) with different forms and designs can be used as heat recovery equipment for the air quality improvement in HVAC systems [33,38,39,40,41,42,43,44,45]. A study showed that the use of a U-shaped HPHE in a hospital could reach 7.64% higher effectiveness or heat recovery of up to 608.45 W [44].

Shahsavar et al. [36] developed an air handling unit design equipped with primary and secondary heat recovery units and could increase the efficiency by 43.75%.

The use of liquid-to-air membrane energy exchangers [46,47], porous metal foam heat exchangers [48], nanofluid as heat transfer fluid [49], phase change material as heat transfer media [50,51,52,53], membrane heat exchanger [54,55,56], and polymer heat exchangers [57,58] affect the energy consumption and recovery and sustainability of HVAC systems positively.

The integration of various energy sources instead of one energy source using multicarrier/multiconverters to cover energy loads is another effective method for reducing energy and cost in HVAC systems. Fabrizio et al. [59] reviewed various integrated designs for HVAC and hot water production systems in buildings based on their advantages and disadvantages and presented their efficiencies for energy recovery. They found that for obtaining optimal results for multienergy systems, the appropriate manufacture data for system design and performance calculation is needed.

The application of ultrasonic energy in HVAC systems for process enhancement or system efficiency improvement, such as air humidification/dehumidification, desiccant regeneration, air purification, heat exchanger, defrosting, and evaporator, has been explored by many researchers [60,61,62,63,64].

Improving the ventilation components in HVAC systems plays an important role in optimizing energy efficiency and improving IAQ [65,66,67]. However, researchers have developed many energy-efficient ventilation methods, such as natural and hybrid ventilation strategies for buildings. Their studies also showed that occupants’ behavior affects energy consumption. The study showed that ventilation was interrelated with various factors, such as indoor and outdoor conditions, building characteristics and application, and occupants’ behavior [65]. Thus, many factors must be considered in designing sustainable ventilation systems.

The effective management of mixed-mode (MM) buildings, which utilize natural and mechanical ventilation systems (hybrid), reduces the energy consumption of HVAC systems in a building; however, the reduction percentage depends on the climate, building type, and evaluation assumptions [68].

**Figure 2:**
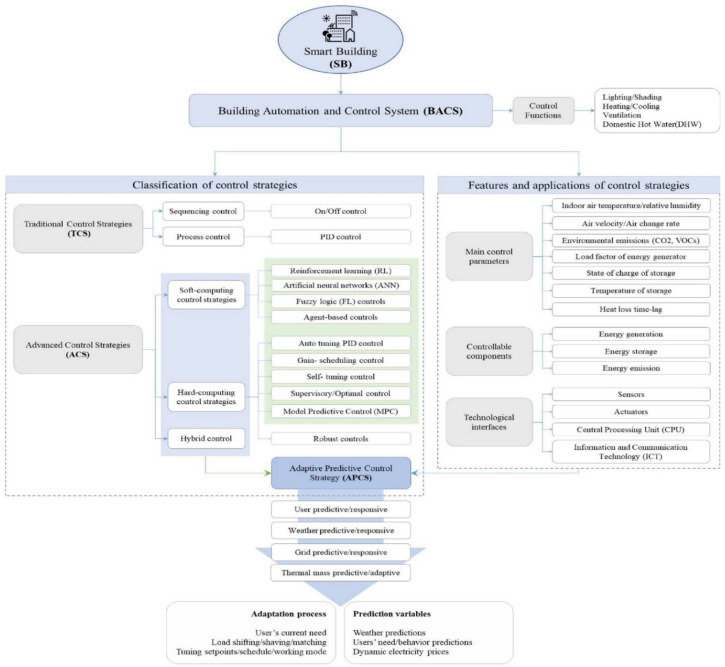
Features, applications, and functions of building automation and control system (BACS) in buildings and HVAC systems [7].

**Figure 3:**
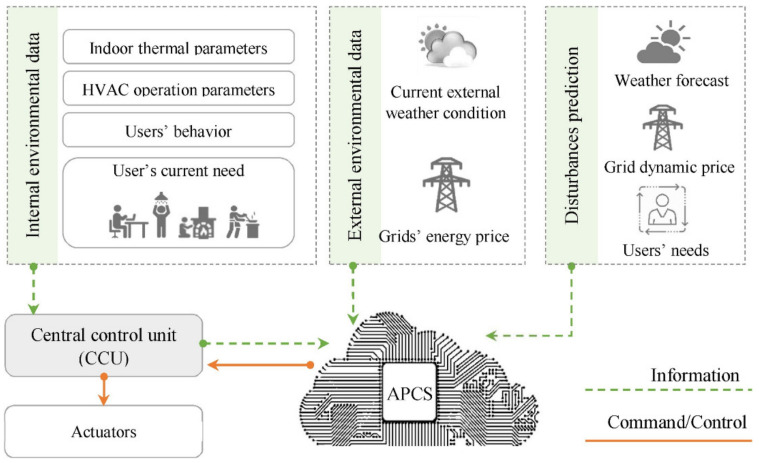
Application of adaptive–predictive control strategies (APCS) as a supervisory control system through a cloud platform [7].

The use of various prediction models to estimate energy consumption based on various parameters in building and HVAC system designs could help us to determine the optimum parameters for reducing energy consumption [69,70,71]. Meanwhile, new integrated designs that implant programmed control systems are very efficient in developing energy-efficient HVAC systems in buildings [33,72,73]. Smart technologies could be predictive/responsive/adaptive against weather, users, grids, and other variables. The use of these smart technologies in buildings and HVAC systems could reduce energy and cost remarkably [7,73] (Figure 2). Figure 3 presents the managing level of adaptive–predictive control strategies in the smart building through a cloud platform. Cloud technology provides additional benefits and functions to the system, and the local controller could still manage the control in case of disconnection. 

**Figure 4:**
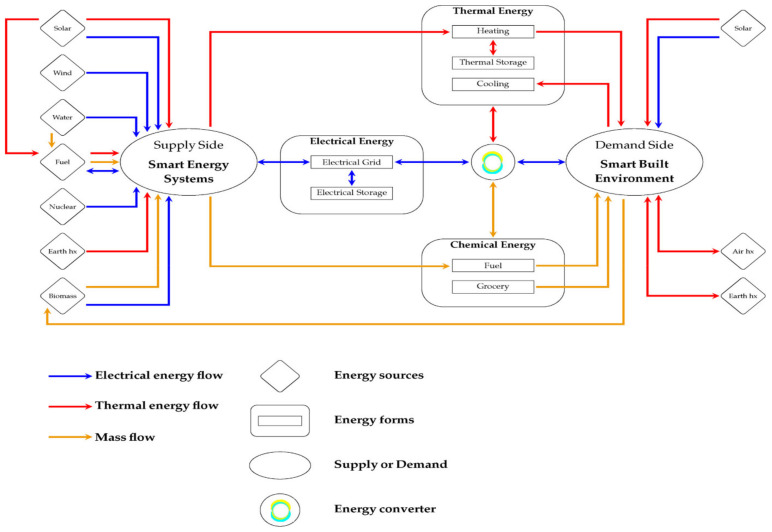
Energy flow charts between the supply side SESs and the demand side SBE [74] (Su 2020).

The progress in artificial intelligence provides a lot of opportunities for developing a smart built environment (SBE) and smart energy consumption. Figure 4 presents the energy flow charts between the supply side of smart energy system (SES) categories and the demand side in the SBE [74]. In the SBE, the use of nonrenewable energy sources reduced greenhouse gas emissions and cost considering the provision of a more sustainable built environment. Su [74] presented some methodologies, system optimizations, and holistic designs for developing SESs and discussed future challenges that require attention. Based on the presented macroscopic view, the fraction of energy can be optimized when the control of the transient fraction of power is adjusted properly. The optimization of the transient fraction of power from different energy forms needs to be considered based on the trade form between the supply side of SESs and the demand side of the SBE.

The use of various decision-making methods, such as multicriteria decision-making (MCDM) methods, could help researchers to find the optimum design for different building types and operation conditions [75,76].

Despite the availability of various modeling techniques developed for improving the control strategy of HVAC systems, finding the optimum model depends on various parameters; moreover, decision makers must be informed about the role, application, advantages, disadvantages, and outcomes of different modeling techniques to find the best option for their application [66,75,77].

Shi et al. [70] investigated a well-validated building simulation model to compare the performance of 12 existing HVAC system strategies in decreasing energy usage, peak demand, and energy cost for decision making in the real world.

## 3. Environment and Society (Thermal Comfort and IAQ)

Spending more than 80% of peoples’ time indoors reveals the importance of IAQ on the health and comfort of occupants [78]. Studies have shown that low IAQ decreases the work performance of occupants [79,80,81].

**Figure 5:**
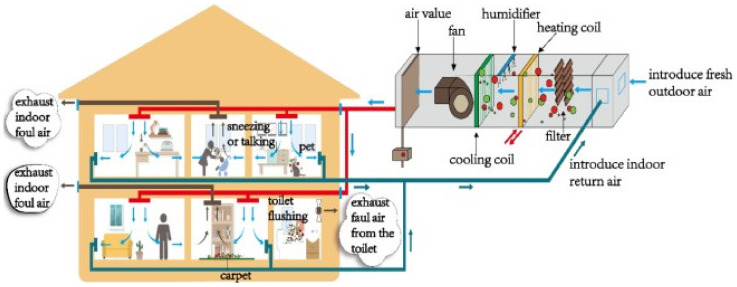
Schematic of microbial contamination in a HVAC system of residential buildings [82].

IAQ in buildings, such as temperature, humidity, airflow, and cleanliness, is directly related to HVAC systems and is considered the respiratory system of buildings [82]. However, improperly designed or operated HVAC systems could contain and develop various microbial contaminations and cause serious health issues for occupants. Recently, various outbreaks of severe epidemic diseases, such as COVID-19, SARS, MERS, and H7N9, and the importance of controlling the spread of various microbial contaminations in HVAC systems, have become critical subjects to consider in the sustainability development of such systems. The microbial contaminations could have been generated in different ways, such as by people (talking/sneezing), pets, carpets, and toilets (Figure 5). These viruses could survive and grow in various HVAC components, such as air ducts, filters, heat exchangers, and fan coils because of their suitable environmental conditions for microbial contaminations [82].

These microbial contaminations diffuse and spread indoors in the form of aerosols or accumulated dust (i.e., suspended particulate matter) with supply airflow and affect the occupants’ health [83]. Microbial contamination with a particle size smaller than 10 μm could be inhaled into the lungs and might cause various respiratory diseases [82]. Therefore, microbial contamination control in HVAC systems is vital for human health and IAQ. Some researchers have used the dehumidifier condensed water to measure volatile organic contaminants and nanoparticles in indoor air [84,85,86].

Liu et al. [82] reviewed various microbial contamination characteristics, and growth, reproduction, and transmission mechanisms caused by HVAC systems to determine effective control methods. They found that filters, heat exchangers (e.g., cooling coil and evaporative condenser), and ventilation ducts are the common components of microbial contamination because their environmental conditions are suitable for their survival and growth.

To prevent microbial contamination in HVAC systems, the control of pollution sources, the regulation of air parameters, and the operation of HVAC systems must be considered completely [82].

Sukarno et al. [33] developed an HVAC system design by adding a heat pipe heat exchanger to remove contaminated air in the airborne infection isolation room in a hospital.

**Figure 6:**
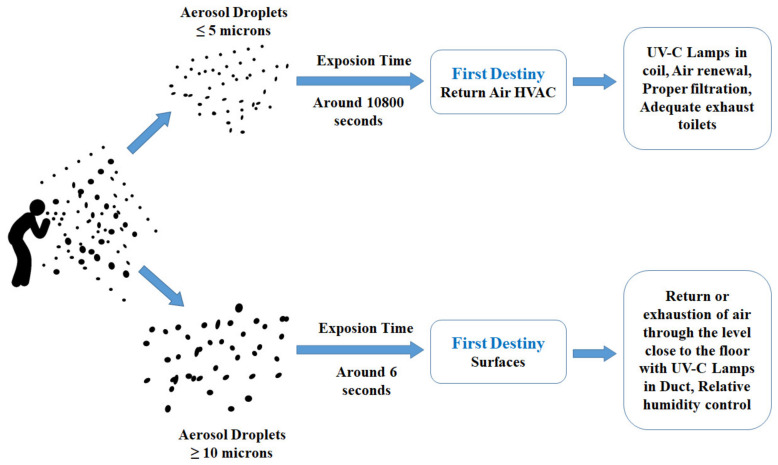
Contagion sources vs. strategies to prevent contagion. Reproduced with permission from [87].

Santos et al. [87] studied various strategies to minimize the risk of COVID-19 infection within the indoor environment through an HVAC system design. They concluded that UV-C lamps, pressure control, air renewal and filtration, restroom actions, and humidity control are effective strategies for minimizing the transmission of airborne infections within the built environment (Figure 6).

Air filtration and purification technologies could modify the IAQ of buildings effectively [88]. However, the influence of various parameters on the performance of these technologies must be considered [89].

Photocatalysis, plasma, UV, microwave sterilization, and physical adsorption are among the common air purification technologies that are combined with HVAC systems to control indoor microbial contaminations [82,90]. However, finding an optimum microbial contamination removal technology needs the consideration of various parameters in HVAC systems. Table 1 presents the comparison of various purification and filtration technologies used in buildings. 

To improve the performance of purification techniques, a combination of strategies, such as plasma combined with photocatalysis and plasma with activated carbon, could be adopted [82].

Paying attention to healthy ventilation is vital when improving the thermal comfort of a building using HVAC systems.

## 4. Water Recovery

As a most treasured resource in the world, the investigation of water sustainability in buildings and HVAC systems is crucial. HVAC systems are considered air–water harvesting systems that liquefy the water vapor available in the air as they condensate; they are also a potential water source [92]. The recovery of condensate has more value, especially in a hot and dry climate which has water scarcity. 

The amount of condensate depends on relative humidity (RH), temperature, air speed, and HVAC system type [93,94,95]. 

Unfortunately, in most cases, the condensate is removed and disposed in sanitary drain [94]. 

Meanwhile, untreated condensate can be the origin of aerosols, which are responsible for microbial contamination in HVAC systems [96]. Water recovery from HVAC systems is also an important issue for water sustainability and building energy recovery, as well as a healthy environment.

**Figure 7:**
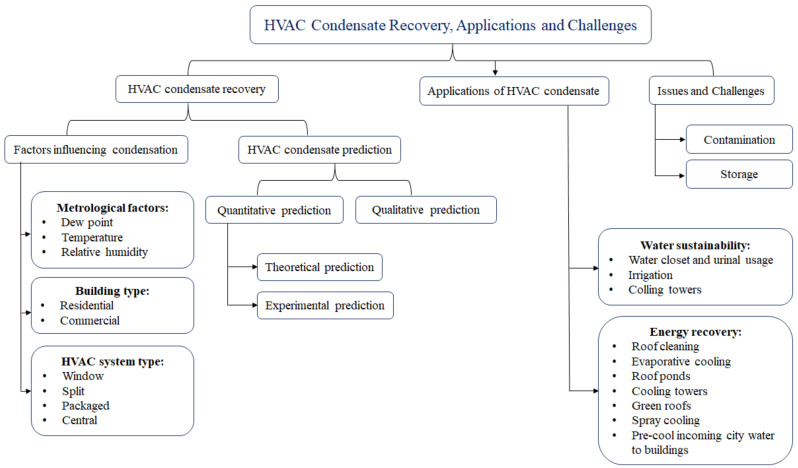
Overview of HVAC condensate recovery, applications, and challenges. Reprinted with permission from [94].

Algarni et al. [94] reviewed various condensate recovery systems in HVAC systems, the potential application of the collected water, and the quality characteristics of the condensate water (Figure 7). They found that planning for a condensate recovery system at the design stage of a building efficiently increased the ease and performance of collecting, storing, and reusing the condensate in the buildings. 

Although many researchers have studied the usage of condensate as potable water because of its high quality (i.e., low mineral and chemical content), the use of proper disinfection methods, such as UV, chlorine, and ozone disinfection, is required to eliminate the potential hazard of microbial contamination [96].

Despite the availability of various studies on the water recovery of HVAC systems, many subjects still need more attention. The application of condensate in evaporative cooling, spray cooling, roof ponds, and green roofs are among the interesting areas that require further research. The separate condensate piping in the plumbing and drainage system in the design stage of HVAC systems could bring more value. More attention to the storage and contamination of condensate based on its end usage plan and optimized water recovery systems as a part of HVAC systems is required [94,97].

Researchers have developed various designs for water and energy recovery from HVAC systems [35,98,99,100,101,102]. Cattani et al. [103] developed an integrated system of HVAC for water production. They also simulated a one-year period of water saving for this integrated system to evaluate its effectiveness. The life cycle assessment showed that rainwater and condensate recovery are sustainable options for meeting the water demands in urban buildings [104,105].

Meanwhile, the development of new designs, such as tri-generation systems [106,107] for combined systems could provide many advantages for building sustainability. 

Yoon et al. [108] used the condensate for water spraying in a portable air conditioner to increase the coefficient of performance (COP) by 16%. 

Tan et al. [109] explored the increase of condensate recovery by coupling a heat pump and membrane distillation (MD). They found that the coupling of MD with the thermoelectric cooler reduced the energy consumption because of the cooling provided by the MD. Meanwhile, the condensate increased because of the improvement in the thermoelectric efficiency. 

## 5. Retrofitting of Existing HVAC Systems (Modification of Old HVAC Systems)

The fact that more than 25% of existing buildings in Europe are more than 70 years old [110,111], 60% of residential building in the US are more than 30 years old [112], and a high percentage of developing countries are currently using low sustainability concepts suggests the importance of considering their retrofitting [113,114]. Retrofitting of existing buildings could decrease the global energy consumption and environmental impact remarkably [6].

Considering the high energy consumption of HVAC systems in buildings, their low sustainability reveals the vital attention required for their modification. This retrofitting could reduce the environmental impacts of the built environment. However, optimum decision making that considers various important parameters, such as energy modeling and assessment, retrofit designs, cost, and risk assessment, is crucial [115,116]. 

Toosi et al. [113] reviewed the life cycle sustainability assessment of studies conducted for building energy retrofitting to determine the life cycle environmental impacts, economic aspects, and the social dimensions of a product, service, or process. They found that the lack of life cycle inventory databases was the main barrier in the life cycle sustainability assessment. Meanwhile, considering other parameters such as future energy mixes, user behavior impacts, macroeconomic parameters, and so on, is important for sustainability assessments. 

The recommended steps for the energy retrofitting of a building [112] are listed as follows:

1. Measurement of HVAC system performance.
2. Identification of potential retrofit alternatives, such as smart controlling systems, upgrading mechanical systems, energy and water recovery, and utilization of renewable energy sources.
3. Establishment of the relation between the investment of retrofitting and the sustainability performance.

Formulation of an optimization model for retrofit investments allocation.

Modification in HVAC systems as a cost-saving opportunity has an important effect on the environmental sustainability of buildings [117]. Monitoring and controlling the HVAC systems is crucial in managing and optimizing the sustainability of HVAC systems. Energy-efficient building designs, considering the important effects of building reflection and insulation on the HVAC system’s performance, and the energy retrofitting of the building’s design, are more effective and easier than upgrading the HVAC systems [117].

According to Patel et al. [117], some useful modification for existing HVAC systems include: 

**Utilizing energy monitoring and control systems (smart systems):** the use of smart monitoring and control technologies in HVAC systems could decrease the energy usage of HVAC systems remarkably [67].

**Discharge air temperature management:** by setting higher discharge air temperatures, the energy for subsequent reheating of cooled make-up air could be reduced and energy could be saved.

**Variable-air-volume systems:** the optimization of air flow into the building, based on its requirements, by adjusting the air flow within the HVAC ductwork could reduce load and save energy.

**Duct leakage repair:** duct leakage is responsible for significant energy waste in HVAC systems. Repairing duct leakage could improve energy usage by 30%.

**Adjustable speed drives:** adjustable speed drives could be used in variable volume air handlers, recirculation fans, chiller pumps, and water pumps to reduce energy consumption.

**Heat recovery systems:** various heat recovery systems could be used in HVAC systems to reduce energy consumption [67].

**Improvement of chiller:** various concepts, such as decreasing the temperature of the condenser water and installing separate high-temperature chillers for process cooling, could improve energy efficiency.

**Fan modification:** selecting the appropriate size and shape of the sheaves of a fan could optimize its efficiency and air flow and decrease energy consumption.

**Exhaust fans improvement:** changing traditional centrifugal exhaust fans with mixed flow impeller exhaust fans could increase efficiency by 25%. They are also cheaper to install and maintain.

**Cooling water recovery:** recycling secondary treated cooling water in chiller systems could improve the sustainability of systems.

**Solar air heating**: in cold climates, the use of solar heating systems, such as a solar wall, not only reduces energy consumption but also provides clean fresh air and reduce emissions.

**Filter’s modification:** utilizing improved filters or filter slots to avoid pressure drops could improve the energy usage in HVAC systems.

Meanwhile, the importance of HVAC system maintenance in terms of energy consumption must not be ignored [117].

## 6. Discussion and Conclusions

Recent pandemic diseases, such as COVID-19, SARS, H7N9, and MERS, and the fact that people spend more than 80% of their time inside buildings show the importance of designing sustainable HVAC systems. Moreover, HVAC systems are the respiratory system of a building, and the potential risk to develop various microbial contaminations that threaten occupant health and work performance shows the urgency to improve its sustainability and performance. 

Meanwhile, the high percentage of existing unsustainable HVAC systems globally shows the vital need for their retrofitting to mitigate environmental, energy, and economical issues. However, controlling and maintaining modified HVAC systems for their sustainability needs careful attention.

However, the optimum design and retrofitting plan of the HVAC system depends on the building types, climatic conditions, and effective parameters for the requested sustainability. Moreover, the decision makers could use decision-making methods to develop optimum plans that consider the various advantages and disadvantages and the potential risk of each option [75,118,119]. Some important recommendations for the better optimization of HVAC systems in buildings are listed as follows:

- Improvement and retrofitting of the building design are very effective for improving the performance of HVAC systems.
- All decision-making parameters for the planning of sustainable HVAC systems, such as occupancy, comfort, health, building type, and cost must be extensively studied.
- Important parameters must be considered in using renewable technologies in HVAC systems to achieve sustainability.
- The use of developed and advanced designs and control strategies in the HVAC systems could increase their sustainability remarkably.
- Improving IAQ and occupants’ health by focusing on ventilation systems and microbial contamination prevention in HVAC systems.
- Water and energy recovery in HVAC systems are crucial parameters for improving sustainability.
- The retrofitting of the existing HVAC system is a vital action in the current situation.
- Optimum plans need to take into account all the advantages and disadvantages of various options while considering the available facilities, conditions, risks, and cost.

## Figures and Tables

**Table 1:** Comparison of different purification and filtration technologies in building environments [82,91].

Technologies	Target	Advantages	Disadvantages	Efficiency
Fiber filtration	Particles, microorganisms	Low cost, convenient installation	Resistance related to the purification efficiency, mid- and high efficiency of high-resistance filters	Can achieve 99.99%
Electrostatic dust removal	Particles, microorganisms	High efficiency and wide range of particle size, small pressure loss	High investment, efficiency decline after dust discharge, easy to breakdown electric field	50% (some only 20%)
Ultraviolet sterilization	Microorganisms	High efficiency, safe and convenient, short reactive time, no residual toxicity, no pollution, low resistance, low cost	Poor dynamic sterilization effect, short service life, UV lamp should be close to the irradiated material, can be influenced by environmental factors, and suspended particles greatly produce secondary pollution	82.90%
Activated carbon adsorption	Nearly all pollutants except biological ones	Wild sources, wide pollutant purifying range, does not easily cause secondary pollution	Saturated regeneration problems, high resistance, poor mineral processing	
Plasma	All indoor pollutants	Wide range of pollutants	Cannot completely degrade pollutants, high energy consumption, and production of by-products (ozone and nitrogen oxides)	66.70%; Cold plasma air filter: 85–98%
Negative ions	Particles, microorganisms	Accelerate metabolism, strengthen cell function, effective to some disease	Produce ozone, cause secondary pollution, dust deposition damages walls	73.40%
Photocatalysis	TVOC, microorganisms, and other inorganic gaseous pollutants	Wide range of purification, mild reaction conditions, no adsorption saturation phenomenon, long service life	Compared with the activated carbon adsorption technology, purification process is slower, easily causes secondary pollution if response is not completed, unable to remove particulate pollutants	75% (some may only 30% or even negative)
Trombe wall	Effective particles (diameter) >10 μm and <0.01 μm	60-year service life	Useless for 0.1–1 μm	99.4% for PM10
Biofilter	Mixture of VOCs	Effective odor control method		Dynamic botanical air filtration system: >33% for toluene and 90% for formaldehyde; integrated biofiltration system: 99%
Microwave sterilization	Bacterial and fungal aerosols	Heating uniformity, rapid sterilization, and no residue combination of thermal and nonthermal effects; under atmospheric pressure, microwaves can induce argon plasma disinfection	Radiation is harmful to human health	30–40% of bacterial and fungal aerosols in the environment can survive for 1.7 min under microwave high-power radiation
